# Supplementary figures and images for: Dominant expansion of CD4+, CD8+ T and NK cells expressing Th1/Tc1/Type 1 cytokines in culture-positive lymph node tuberculosis
Source: PLoS One. 2022 May 26;17(5):e0269109. doi: 10.1371/journal.pone.0269109 (PMC9135291; doi:10.1371/journal.pone.0269109)

Supplementary Figure 1

A

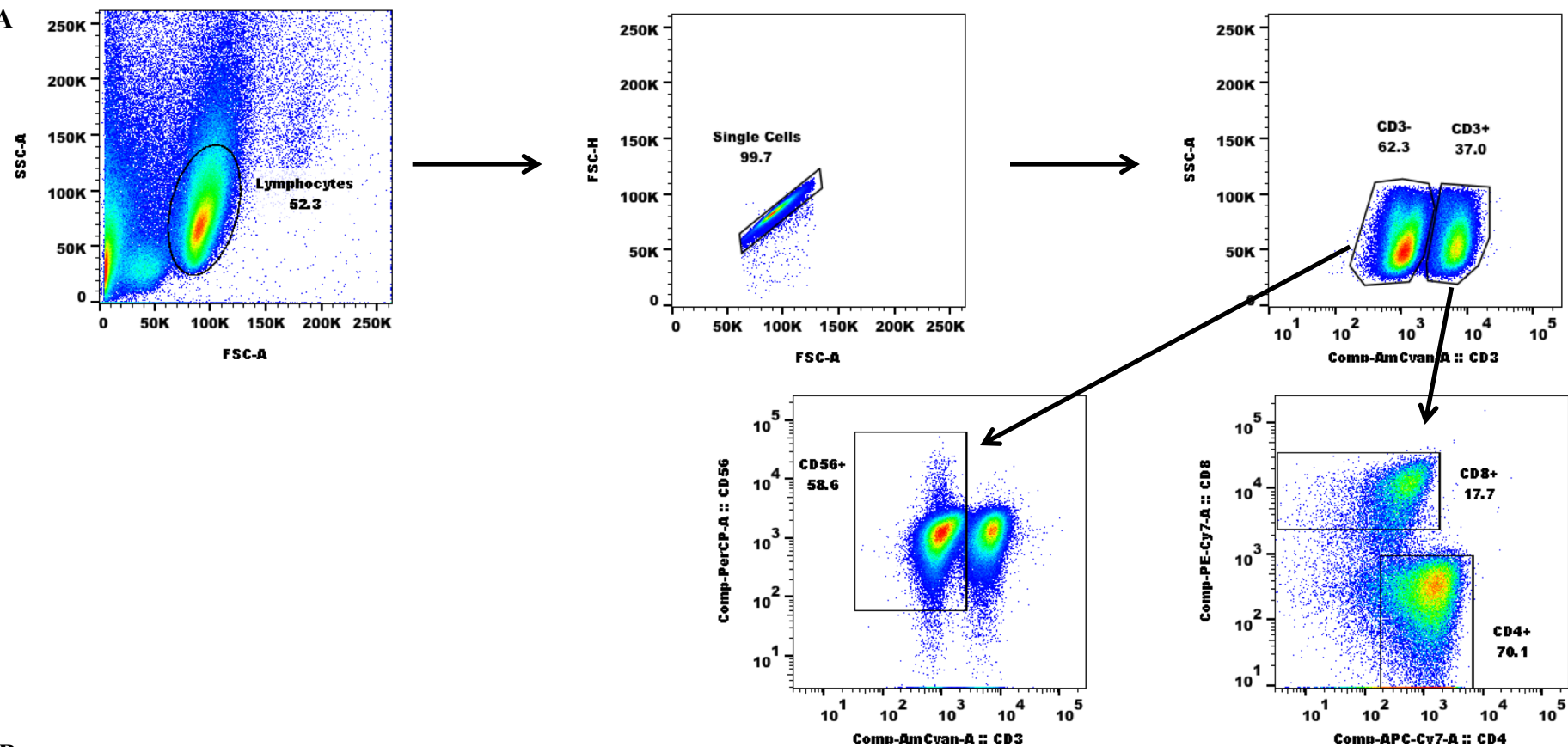

B

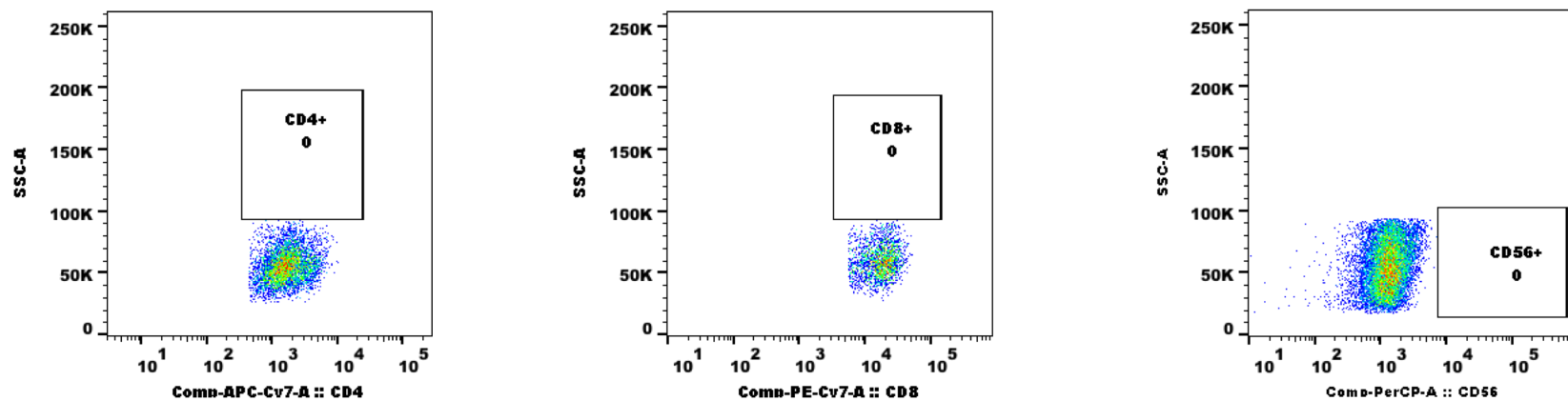

Supplement: S1 Fig — (A) Lymphocytes are gated on LN sample and further gated on single cells. From single cells CD3+ T cells and NK (CD3-CD56+) cell population were gated. CD3+ T cells were further gated for CD4+ and CD8+ T cells. (B) FMO population of CD4+, CD8+ T cells and NK cells. (PDF) [file pone.0269109.s001.pdf]

Supplementary Figure 2

Th1 cytokines

Th17 cytokines

Cytotoxic markers

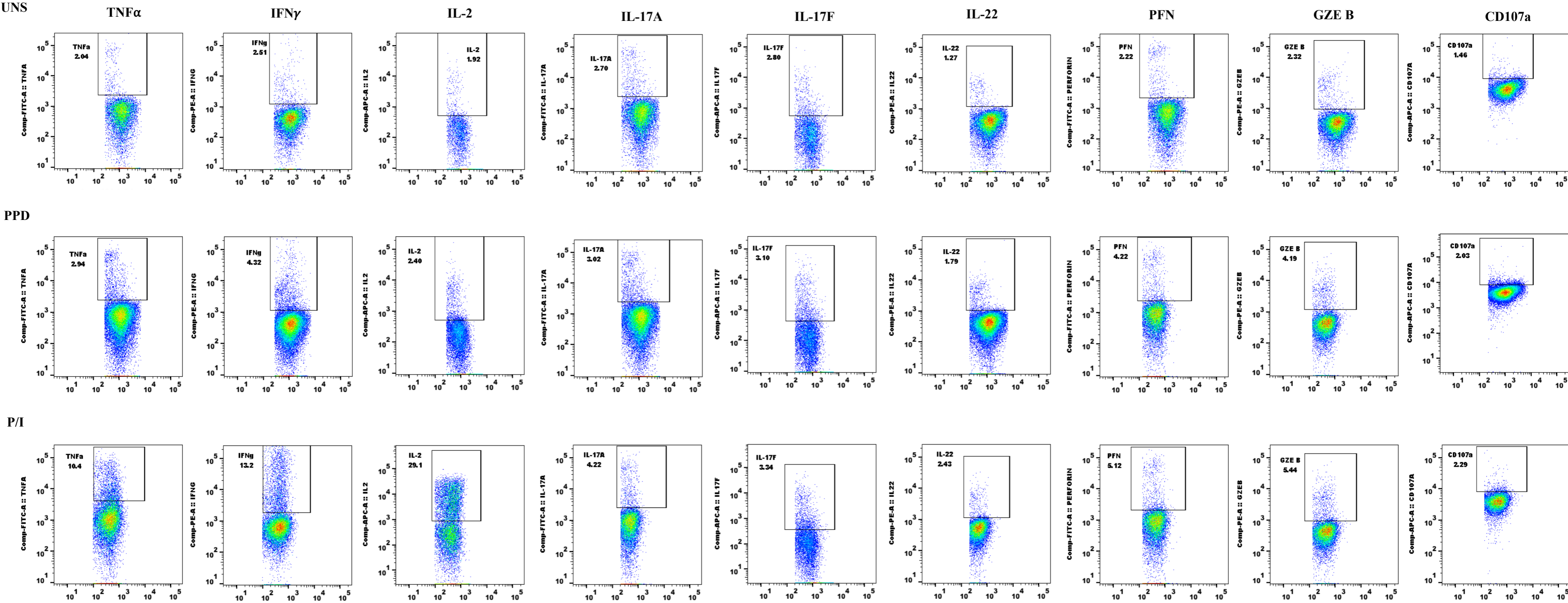

Supplement: S2 Fig — (A) Th1 (IFNγ, TNFα, IL-2) cytokines, (B) Th17 (IL-17A, IL-17F, IL-22) cytokines, (C) cytotoxic (PFN, GZE B, CD107a) markers upon UNS, Mtb (PPD) antigen stimulation and positive antigen (P/I) control stimulation. (PDF) [file pone.0269109.s002.pdf]

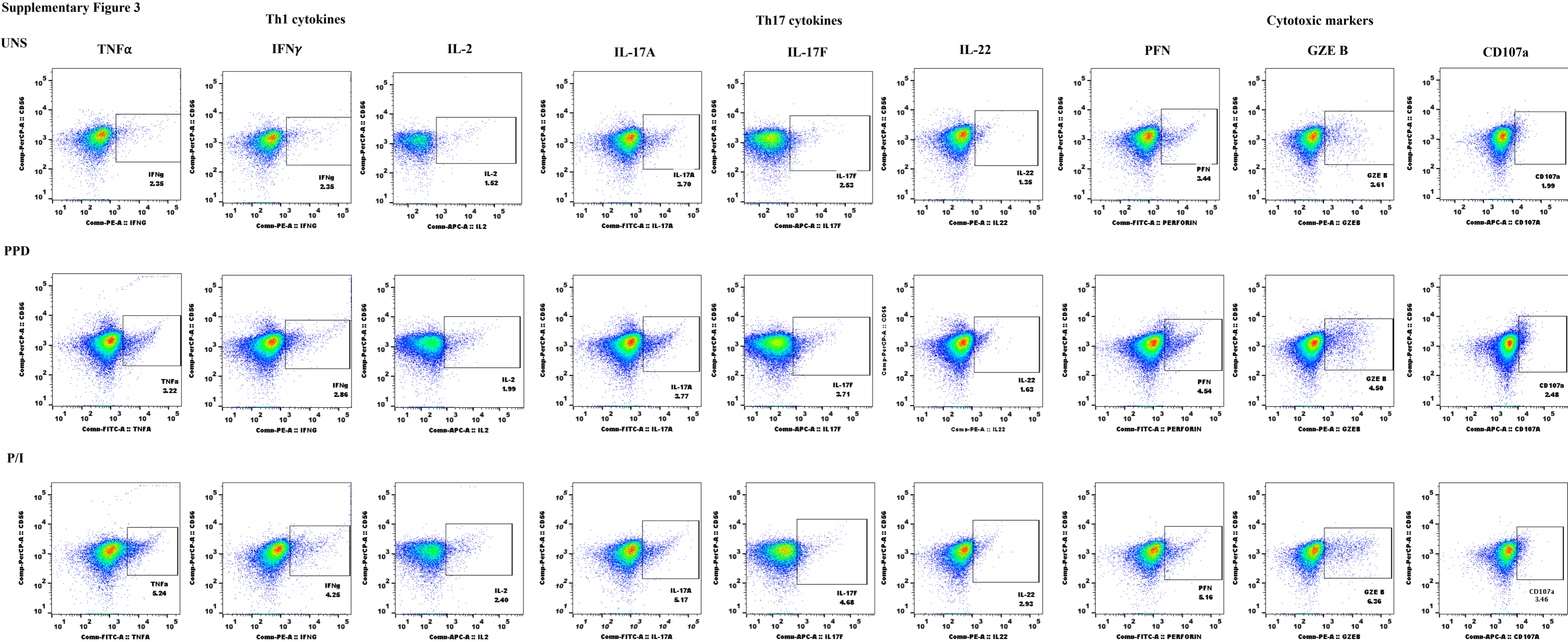

Supplement: S3 Fig — (A) Th1 (IFNγ, TNFα, IL-2) cytokines, (B) Th17 (IL-17A, IL-17F, IL-22) cytokines, (C) cytotoxic (PFN, GZE B, CD107a) markers upon UNS, Mtb (PPD, CFP-10 PP) antigen stimulation and positive antigen (P/I) control stimulation. (PDF) [file pone.0269109.s003.pdf]
