# Supplementary material for: Dominant expansion of CD4+, CD8+ T and NK cells expressing Th1/Tc1/Type 1 cytokines in culture-positive lymph node tuberculosis
Source: PLoS One. 2022 May 26;17(5):e0269109. doi: 10.1371/journal.pone.0269109 (PMC9135291; doi:10.1371/journal.pone.0269109)
Supplement: S1 Table — (DOC) [file pone.0269109.s004.doc]

**Supplementary Table 1. Antibodies panel used in the study.**

| **Panel*** | **FITC** | **PE** | **APC** | **APC Cy-7** | **PE CY-7** | **PERCP** | **AMCYN** |
| --- | --- | --- | --- | --- | --- | --- | --- |
| FMO | - | - | - | CD4  (1 μl) | CD8  (1 μl) | CD56  (1 μl) | CD3  (3 μl) |
| I | TNFα  (5 μl) | IFNγ  (1 μl) | IL-2  (2 μl) | CD4  (1 μl) | CD8  (1 μl) | CD56  (1 μl) | CD3  (3 μl) |
| II | IL-17A  (5 μl) | IL-22  (2 μl) | IL-17F  (2 μl) | CD4  (1 μl) | CD8  (1 μl) | CD56  (1 μl) | CD3  (3 μl) |
| III | Perforin  (5 μl) | GZE B  (2 μl) | CD107a  (2 μl) | CD4  (1 μl) | CD8  (1 μl) | CD56  (1 μl) | CD3  (3 μl) |

| **Antibody name & company** | **clone** | **Catalogue/product number** |
| --- | --- | --- |
| TNFα (Mouse anti-Human, BD) | 6401.111 | 340511 |
| IFNγ (Mouse anti-Human, BD) | B27 (RUO) | 562016 |
| IL-2 (Monoclonal antibody, eBioscience™) | MQ1-17H12 | 17-7029-82 |
| IL-17A (anti-human, Miltenyi Biotec) | CZ8-23G1 | 130-120-410 |
| IL-22 (Monoclonal antibody, R&D) | 22URTI | IC7821P |
| IL-17F (Monoclonal Mouse IgG2B, R&D) | 197301 | IC13351A |
| Perforin (anti-Human, BD Pharmingen) | +BD7238 | BDB556577 |
| GZE B (Monoclonal Antibody, eBioscience™) | GB11 | 12-8899-41 |
| CD107a (Mouse anti-Human, BD) | H4A3 | BDB641581 |
| CD3 (Mouse anti-Human, BD) | SK7 (also known as Leu-4) | 339186 |
| CD4 (Mouse anti-Human, BD) | SK3 (also known as Leu3a) | 341095 |
| CD8 (Mouse anti-Human, BD) | SK1 | BDB335787 |
| CD56 (Monoclonal Antibody, eBioscience™) | TULY56 | 46-0566-42 |

FITC (Fluorescein isothiocyanate); PE (phycoerythrin); APC (Allophycocyanin); PerCP (Peridinin chlorophyll protein)

*Panel I - Th1/Tc1/Type1 cytokines; Panel II - Th17/Tc17/Type17 cytokines; Panel III- Cytotoxic markers.
