# Supplementary material for: Dominant expansion of CD4+, CD8+ T and NK cells expressing Th1/Tc1/Type 1 cytokines in culture-positive lymph node tuberculosis
Source: PLoS One. 2022 May 26;17(5):e0269109. doi: 10.1371/journal.pone.0269109 (PMC9135291; doi:10.1371/journal.pone.0269109)
Supplement: Attachment — Submitted filename: Comments.docx [file pone.0269109.s005.docx]

In general, the manuscript needs significant review before submission. Authors should proof read and correct all grammar before any submission. There is also lack of consistency in the abbreviations used throughout the manuscript. Abbreviations used to indicate LNTB+ and – should be consistent. Methods section lacks details of protocols and reagents used. All the figures need to follow consistent formatting. Unfortunately, the manuscript is not up to standard of publishing.

Abstract:

1. Abstract should not be in split into sections. Please combine background, methods, results and conclusions together and submit as one paragraph.
2. Line 30: It should be (LNTB-)

Introduction:

1. Introduce what LN stands for e.g. lymph node (LN) before using abbreviation. If you’re using LN for lymph node throughout manuscript, please use this abbreviation.
2. Briefly explain the significance of your research and why it’s important to distinguish between LNTB+ and –
3. Line 95-97, TBL LNs are you referring to LNTB+? Avoid using TBL and LNTB+ interchangeably. Stick to one term
4. Line 98- provide e.g. of increased cytokines and decreased cytokines. Otherwise your statement is too vague.

Methods:

Lymph node isolation, culture and antigen stimulation.

1. How were the lymph nodes extracted? Were cells purified before use? If so what component? Or were they used directly?
2. Where the lymph nodes used for culture immediately after removal or were they stored and used later? Please indicate these steps.
3. Authors need to mention concentrations of each stimulation used.
4. Please detail all exact protocols used.

Intracellular staining of lymph nodes

1. Make heading of this section “Extra cellular and intra cellular staining of lymph nodes”.
2. Please provide list of antibodies used in the form of a supplementary table, with cat#, manufacturer, volume of antibody used in final volume.
3. Provide detail protocols, i.e. step by step on the antibody staining procedure.
4. Mention the manufacturer names of the permeabilization buffer and media used.
5. Provide recipes of cell culture media and any and all buffers with their respective manufacturer names.
6. What is the FlowJo version number?

Results:

Figure 1: Use different colours to indicate two cohorts (black/red). Axis labels are too small and difficult to read. Please increase text size. Use consistent labeling to indicate LNTB+/- not cult+ and cult – on figures. This is applicable to entire manuscript where authors are not consistent with using the abbreviations.

The same comments apply to figure 2 and 3.

Supplementary figures 1, 2, 3: Use same format for gating strategy. If using pseudo colour, stick to pseudo colour if not stick to contour plots for all three. Increase font size of the labels and axis. Clearly mark in each plot the population of cells you’re referring to and use consistent formatting. Use the same formatting for every figure. Keep it consistent.

Figure 2 and 3: Show one representative plot from an unstimulated for negative and one positive control for each cytokine to give readers an idea of where the gates were set. You do not need to show plots for each stim condition.

Discussion:

1. Line 303-305: How did you arrive to this conclusion? Where are the ref papers to back up your claim?
2. Line 314-316: Ref?
3. Line 324-326: ref?
