# Supplementary material for: Dominant expansion of CD4+, CD8+ T and NK cells expressing Th1/Tc1/Type 1 cytokines in culture-positive lymph node tuberculosis
Source: PLoS One. 2022 May 26;17(5):e0269109. doi: 10.1371/journal.pone.0269109 (PMC9135291; doi:10.1371/journal.pone.0269109)
Supplement: Attachment — Submitted filename: Reply to Review Comments Revision II.doc [file pone.0269109.s008.doc]

Review Comments to the Author

Reviewer #1: Overall there is a definite improvement in the new draft. However, authors will benefit from another round of proof reading before the final submission.

**Response: Thank you for the valuable comments on our manuscript.**

211 T cells expressing Tc1 and Tc17 (expect IL-17F) cytokines and cytotoxic marker frequencies: change expect to except

**Response: We apologize for the spelling error and now we changed expect to except.**

213 Finally, upon P/I antigen stimulation, CD4+ 213 T cells 214 expressing Tc1 and Tc17 cytokines and cytotoxic marker frequencies are not significantly different between the study groups (Figure 2F).

This is CD8 and not CD4.

**Response: We apologize for the typographical mistake and now we changed CD4 to CD8.**

Figure 1-3 would benefit from a legend to state which stimulation condition is demonstrated in each row of graphs. This will help reader easily navigate and identify plots.

**Response: As suggested by the reviewer now we included the legends for the stimulation in figures 1-3.**

Discussion

As suggested by the reviewer now we have included the reference number in the line number (now the line number is 413-415). Discussion section Line 324-326: ref?

**Response: We have included the reference (reference number 24) in line number 324-326.**

**24. Kumar NP, Sridhar R, Hanna LE, Banurekha VV, Jawahar MS, Nutman TB, Babu S. Altered CD8(+) T cell frequency and function in tuberculous lymphadenitis. Tuberculosis (Edinb). 2014; 94(5):482-93. doi: 10.1016/j.tube.2014.06.007.**

As suggested by the reviewer now we have included the reference number in the line number (now the line number is 418, 437-439). Discussion section

Line 413-415, 418, 437-439 does not indicate to the correct lines in this version of the draft. Please indicate the correct line numbers for reviewers in the future.

**Response: We apologize and now we have included the correct line numbers (line numbers 292-294 and 302-304 in the clean version of the manuscript) along with the references.**

**24. Kumar NP, Sridhar R, Hanna LE, Banurekha VV, Jawahar MS, Nutman TB, Babu S. Altered CD8(+) T cell frequency and function in tuberculous lymphadenitis. Tuberculosis (Edinb). 2014; 94(5):482-93. doi: 10.1016/j.tube.2014.06.007.**

**8. Kathamuthu GR, Moideen K, Sridhar R, Baskaran D, Babu S. Enhanced mycobacterial antigen-induced pro-inflammatory cytokine production in lymph node tuberculosis. Am J Trop Med Hyg. 2019; 100(6):1401-1406. doi:10.4269/ajtmh.18-0834**

**27. Kathamuthu GR, Kumar NP, Moideen K, Sridhar R, Baskaran D, Babu S. Diminished type 1 and type 17 cytokine expressing - Natural killer cell frequencies in tuberculous lymphadenitis. Tuberculosis (Edinb). 2019; 118:101856. doi: 10.1016/j.tube.2019.101856.**
